# Systematic Review and Meta-Analysis of the Effectiveness of Teacher Delivered Interventions for Externalizing Behaviors

**DOI:** 10.1007/s10864-022-09491-4

**Published:** 2022-09-03

**Authors:** R. Aldabbagh, C. Glazebrook, K. Sayal, D. Daley

**Affiliations:** 1 Division of Psychiatry and Applied Psychology, School of Medicine, University of Nottingham, Nottingham, UK; 2 Centre for ADHD and Neurodevelopmental Disorders Across the Lifespan, Institute of Mental Health, Nottingham, UK; 3 Special Education Department, Jeddah University, Jeddah, Saudi Arabia

**Keywords:** Teacher training, Teacher intervention, Child behavior problems, Externalizing behaviors, Attention deficit hyperactivity disorder, ADHD

## Abstract

This systematic review and meta-analysis explores the effectiveness of teacher interventions supporting children with externalizing behaviors based on teacher and child outcomes. A systematic search was conducted using 5 electronic databases. From 5714 papers, 31 papers that included interventions delivered directly to teachers and aimed to benefit either teachers and/or children with externalizing behaviors were included. The review focused on qualified teachers working with children aged 2–13. The results of the current meta-analysis revealed a positive effect of teacher intervention on teacher and child outcomes, including the increased use of teacher-appropriate strategies, as well as significant and moderate improvements in teacher–child closeness, and small reductions in teacher–child conflict. For child outcomes, the interventions reduced externalizing behavior problems and ADHD symptoms and enhanced prosocial behavior. Only one fully blinded analysis for conduct problems was possible and revealed a moderate but significant reduction in favor of intervention. These findings provide evidence to support the role of teacher interventions for both teachers and children with externalizing behaviors. Future research should include more PBLIND measurements so that MPROX findings can be confirmed. More research should be done to evaluate the influence of teacher interventions on teachers’ well-being.

## Introduction

Externalizing behaviors, including conduct problems and the symptoms associated with attention deficit hyperactivity disorder (ADHD), account for about 7% of school-based problems in childhood (Polanczyk et al., 2014). Children with externalizing behaviors can be challenging to teach because they display higher levels of developmentally inappropriate behaviors like hyperactivity, inattention, disobedience, impulsivity, and tantrums (Doepfner et al., 2004). Children who exhibit externalizing behaviors are often a focus of classroom disruption (Daley et al., 2014) and also experience disrupted peer relationships (Lewis et al., 2016). Teachers’ well-being can also be influenced by the impact of children’s externalizing behaviors on classroom management (Brill & McCartney, 2008), which highlights both the direct and indirect impact of these behaviors.

Externalizing behaviors in children are often associated with greater levels of peer rejection and more difficulty with friendship formation (Rubin et al., 2018), as well as challenges with teamwork, peer interaction, and sharing (Ettekal & Ladd, 2014). Children who engage in externalizing behaviors often struggle to interact with teachers (Williford et al., 2017); fail to follow appropriate teacher instructions or complete work on time (DuPaul & Stoner, 2014). They also display frequent tantrums and/or outbursts (Dupaul et al., 2001). Consequently, implementing educational programming with children who display externalizing behavior is a challenging task for teachers (Williford & Shelton, 2014).

Providing teachers with appropriate behavioral strategies to help manage externalizing behaviors more effectively is vital for the children themselves, as well as overall classroom management (Daley et al., 2014), and teachers’ well-being (Aloe et al., 2014). Previous research studies have shown that parenting interventions that help parents to modify the home environment can reduce children’s level of externalizing behaviors at home and enhance social development (Webster-Stratton, 2011). Buchanan-Pascall et al. (2018) conducted a systematic review and meta-analysis that investigated the efficacy of parent-mediated intervention for children with externalizing behaviors and concluded that 80% of studies confirmed the efficacy of parent-mediated interventions, thus supporting the parent’s role in reducing externalizing behaviors (Buchanan-Pascall et al., 2018). However, although numerous studies and meta-analyses have explored parent-mediated interventions, there is little research on teacher-mediated interventions despite evidence that shows the impact of externalizing behaviors on teachers’ well-being (Aloe et al., 2014) and self-efficacy (Collie et al., 2012); both of which can have an immediate impact on children in the classroom (Miller et al., 2017).

Teachers often struggle to manage children with externalizing behavior (Herman et al., 2017) and classroom disruption can interfere with the teaching process (Savage et al., 2017). For example, Arbuckle and Little (2004) asked 96 teachers to report the types of challenging behavior experienced by teachers and their confidence in utilizing behavior management strategies in the classroom; their findings showed that child aggression and hyperactivity were teachers’ greatest concerns. Arbuckle and Little also found an association between lower teacher confidence and higher levels of hyperactivity and non-compliance in male students. Arbuckle and Little’s findings are consistent with other studies that have shown that lower teacher confidence levels are associated with higher levels of difficulty in terms of teachers’ ability to teach and manage difficult students (Stephenson et al., 2000).

Difficult-to-manage student behavior in the classroom can also impact the teacher–child relationship (TCR). Daley et al., (2005) and McGrath and Van Bergen (2017) both explored TCR using measures of expressed emotion (EE) to gauge warmth and criticism expressed by teachers toward children with externalizing behavior. Both studies found that teachers directed higher levels of criticism and lower levels of warmth toward students who displayed externalizing behaviors than to matched controls. These studies focused exclusively on the impact that disruptive pupils may have on TCR (Daley et al., 2005; McGrath & Van Bergen, 2017). Since parental EE has also been shown to correlate with negative parent–child interaction (Daley et al., 2005; Tompson et al., 2015), it is plausible that teacher EE may also be a proxy marker for teacher–pupil interaction. Therefore, higher teacher warmth and lower teacher criticism may help to reduce the expression of externalizing behaviors in the classroom, although this has not yet been empirically examined. The current systematic review and meta-analysis therefore aims to explore the effectiveness of teacher-delivered interventions in supporting children who display externalizing behaviors based on child and teacher outcomes.

## Methods

### Search Strategy

The researchers conducted the search on February 5, 2018, and it was refreshed on September 27, 2018, April 19, 2020, and July 16, 2021, using the following electronic databases: Ovid (Embase 1974-present, MEDLINE 1980-present, PsycINFO, PsycARTICLES), and Web of Science database. To make the search more comprehensive, the researchers conducted backward and forward citation searching using Google Scholar (Higgins et al., 2019; Polanin & Pigott, 2015). See PROSPERO registered protocol number (CRD42018095476) for more details.

### Inclusion Criteria

The authors limited the scope of the published trials based on Cochrane group recommendations (Higgins et al., 2019). The current meta-analysis included only peer-reviewed, randomized control trials (RCTs) published in English and focused on externalizing behaviors in childhood. The current systematic review and meta-analysis focused on qualified teachers working with children aged 2–13 years and studies that measured teacher outcomes and/or child problem behavior. The current analysis included children who exhibited high levels of externalizing behaviors (ADHD symptoms or conduct problems) based on teacher reports. All studies had to include behavioral interventions delivered specifically to teachers and aimed to benefit either teachers and/or children with externalizing behaviors. The authors excluded studies that measured only teachers’ knowledge; comparison control conditions could include waiting lists, treatment as usual, or alternative treatment, while trials could include either child and/or teacher outcomes. The researchers also excluded studies that included individuals with Autism Spectrum Disorder (ASD) since the intention was to focus on externalizing behaviors and individuals displaying high levels of symptoms of behavioral disorders (e.g., ADHD; oppositional defiant disorder [ODD]; conduct disorder).

### Study Selection

**Fig. 1:**
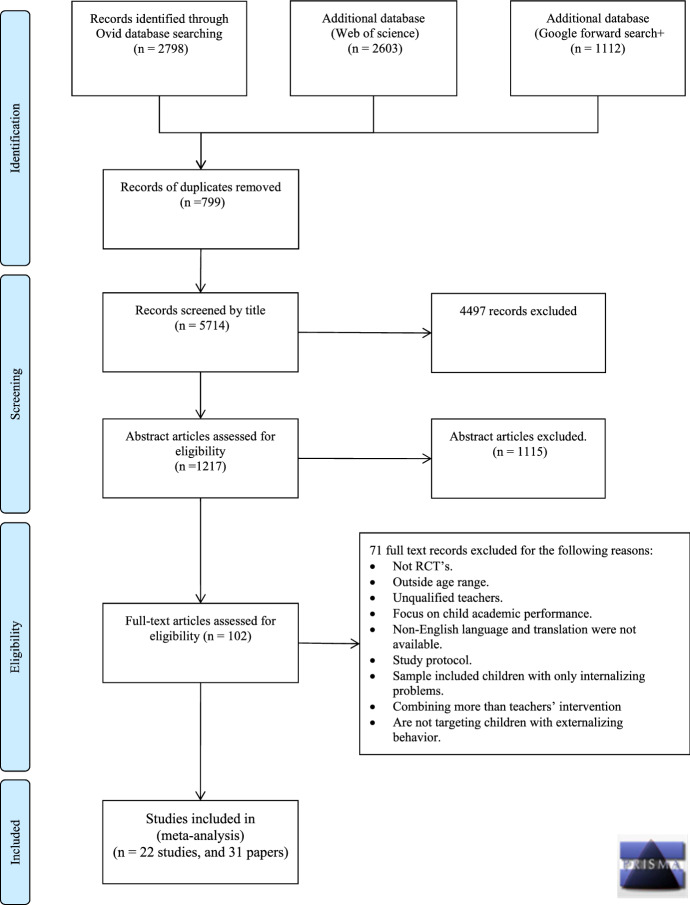
PRISMA diagram

The PRISMA protocol (Moher et al., 2009) informed the current systematic review and meta-analysis. Figure 1 shows a flowchart describing the process for study selection; the retrieved references were screened by title and abstract independently and blindly double-coded for eligibility. The authors resolved any disagreements between them and reviewed full-text articles for eligibility (see Fig. 1).

Thirty-one studies met the inclusion criteria, but only 22 of them contributed to the meta-analysis as nine studies did not provide data that could be used in the meta-analysis (see Table 1); nevertheless, the authors included them in the systematic review by describing the interventions and identifying the study characteristics, as shown in Tables 2 and 3. Some of the included studies employed the same sample size yet produced different outcomes. The authors accounted for this when computing the total number of students and teachers to avoid doubling the figures.

**Table 1:** Overview of Studies Included in the Systematic Review and the Meta-analysis (Studies that contributed to the analysis are shown in bold)

#	Study references
**1**	*(Baker-Henningham et al., 2009a**)**
**2**	*(Baker-Henningham et al., 2009b**)**
**3**	*(Baker-Henningham et al., 2012**)**
**4**	*(Baker-Henningham & Walker, 2018**)**
**5**	**(Bloomquist et al.,** 1991**)**
**6**	**(Caldarella et al.,** 2018**)**
**7**	**(Conroy et al.,** 2015**)**
8	(Conroy et al., 2021)
**9**	**(Corkum et al.,** 2019**)**
**10**	**(Downer et al.,** 2018**)**
**11**	**(Gonzales-Ball & Bratton,** 2019**)**
**12**	**(Hickey et al.,** 2017**)**
13	*(Hoogendijk et al., 2018)
**14**	*(Hoogendijk et al., 2020**)**
**15**	**(Hutchings et al.,** 2013**)**
**16**	*(LoCasale-Crouch et al., 2018**)**
**17**	*(Williford et al., 2017**)**
18	(McCullough et al., 2021)
19	(Owens et al., 2017)
**20**	**(Reinke et al.,** 2014**)**
**21**	**(Spilt et al.,** 2012**)**
**22**	**(Stoiber & Gettinger,** 2011**)**
**23**	**(Sutherland et al.,** 2018a**)**
24	*(Conroy et al., 2019)
25	*(Conroy et al., 2018)
26	*(Sutherland et al., 2018b)
**27**	*(Sutherland et al., 2020**)**
**28**	*(Vancraeyveldt et al., 2015a**)**
29	*(Vancraeyveldt et al., 2015b)
30	*(Veenman et al., 2017)
31	*(Veenman et al., 2019)

*The following papers between brackets are one study using the same sample but reporting different outcomes

(Baker-Henningham & Walker, 2018; Baker-Henningham et al., 2012), (Baker-Henningham et al., 2009a, 2009b), (Conroy et al., 2018, 2019; Sutherland et al., 2018a, 2018b), (Hoogendijk et al., 2018, 2020), (LoCasale-Crouch et al., 2018; Williford et al., 2017), (Vancraeyveldt et al., 2015a, 2015b), (Veenman et al., 2017, 2019)

**Table 2:** Summary of characteristics of studies included in the systematic review and the meta-analyses

Trial	Children selection	Selected by	Design	Control condition	Teachers Number I C	Teaching experience mean I C	Female percentage	Students Number I C	Age range in years	Percentage of boys
Baker-Henningham et al. (2009a, 2009b)	Children were selected using Strengths and Difficulties Questionnaire (SDQ)	Teachers	Four-arm RCT	AT	15	14	N/A	69	3–5	65.15%
					12	12		66		
Baker-Henningham et al. (2012), Baker-Henningham and Walker, (2018)	Children were selected using ICD-10 Diagnostic criteria for research on conduct disorder	Interview with the classroom teacher using questionnaire	Four-arm RCT	TAU	37	12	94.55%	113	3–6	61.35%
					36	13		112		
Bloomquist et al. (1991)	A screening method is known as multistage identification, to diagnose ADHD	Teachers	Three-arm RCT	WLC/AT	N/A	N/A	N/A	16	6–6	69.00%
								16		
Caldarella et al. (2018)	Children at risk of behavioral problems were selected using SSBD	Teachers	Two-arm RCT	TAU	160*	9	95.00%	177	6–12	50.00%
						9		134		
Conroy et al. (2015)	Children were selected if they are at risk of emotional behavioral problems EBD using Early Screening Project (ESP)	Teachers	Two-arm RCT	TAU	26	15	100.00%	66	3–5	63.75%
					27	13		64		
Conroy et al. (2021)	Children were identified at risk for an (ESP)	Teachers	Two-arm RCT	AT	10	6	95.00%	18	3–5	50.97%
					12	18		21		
Corkum et al. (2019)	Students were previously diagnosed with ADHD	physician, psychologist	Two-arm RCT	WLC	28	12	91.40%	28	6–12	88.05%
					30	15		30		
Downer et al. (2018)	The top two boys and one girl who scored high on ADHD and ODD rating by ADHD and ODD Rating Scale DuPaul	Teachers	two-arm RCT	TAU	22	19	100.00%	66	2.5–5.5	65.00%
					22	19		66		
Gonzales-Ball & Bratton, (2019)	Children who exhibiting disruptive behavior were chosen	Teachers	Two-arm RCT	AC	11	7	85.00%	11	3–4	74.20%
					12	7		9		
Hickey et al. (2017)	Students who scored above the cutoff > 12 SDQ	Teachers	Three-arm RCT	WLC	11	9	96.00%	33	5–6	57.00%
					11	11		30		
Hoogendijk et al., (2018, 2020)	Students who scored high in SDQ and STRS conflict	Teachers	Two-arm RCT	TAU	53	12	76.70%	53	8–12	77.00%
					50	12		50		
Hutchings et al. (2013)	Students who scored high in SDQ were selected	Teachers	Two-arm RCT	TAU	6	8	100.00%	13	3–7	66.60%
					6	11		14		
LoCasale-Crouch et al. (2018) and Williford et al. (2017)	The top two boys and one girl who scored high on ADHD and ODD rating by ADHD and ODD Rating Scale DuPaul	Teachers	Three-arm RCT	TAU	66	8	94.80%	137	3–5	66.55%
					61	9		124		
McCullough et al. (2021)	Children were included if they were at risk of having externalizing behavior based on ESP and SSBD	Teachers	Two-arm RCT	TAU	14	6	100.00%	25	8–9	82.00%
					12	6		20		
Owens et al. (2017)	Children were included if they meet diagnostic criteria for ADHD or were at risk of ADHD based on DSM-IV	Teachers selected children, then coulure’s checked eligibility with parents	Two-arm RCT	AT	29	14	93.00%	27	6–12	77.60%
					27	14		29		
Reinke et al. (2014)	Children were chosen by Observation of Classroom Adaptation– Checklist (TOCA-C): the top 15% of children with disruptive behavior were selected	Teachers	Two-arm RCT	AT	34*	N/A	91.00%	23	3–8	69.50%
								23		
Spilt et al. (2012)	Children with externalizing behaviors above median level on Preschool Behavior Questionnaire (PBQ)	Teachers	Two-arm RCT	AT	16	13	N/A	32	3–9	70.00%
					16	13		32		
Stoiber and Gettinger (2011)	Children were selected by teachers as having challenging behaviors	Teachers	Three-arm RCT	TAU	35	14	96.00%	33	4–7	76.00%
					35	11		33		
Sutherland et al., (2018a, 2018b) and Conroy et al., (2018, 2019)	Children were identified at risk for an EBD using (ESP)	Teachers	Two-arm RCT	TAU	113	11	90.50%	110	3–5	64.70%
					110	13		98		
Sutherland et al. (2020)	Children were identified as preforming externalizing behaviors based on Systematic Screening for Behavior Disorders SSBD	Teachers	Two-arm RCT	TAU	14	6	100.00%	25	7–8	82.50%
					16	6		20		
Vancraeyveld et al. (2015a, 2015b)	Boys with high level of externalizing behavior were selected using the (PBQ)	Teachers	Two-arm RCT	TAU	89	N/A	98.30%	89	3–6	100.00%
					86			86		
Veenman et al., (2017, 2019)	Children with ADHD symptoms based on the Disruptive Behavior Disorders Rating Scale (DBDRS) were chosen, followed by an interview with the teachers to confirm eligibility of the child based on the DSM-IV-TR	Teachers and cotches	Two-arm RCT	TAU	N/A	N/A	N/A	58	6–13	84.00%
								56		

AT, alternative treatment; C, Control; I, Intervention; TAU, treatment as usual; WLC, waiting list control; AC, active control

*These two studies provided the total number of teachers in the trial as they have only child outcomes

**Table 3:** Describing the intervention used in the included trials

Trial	Intervention type	Intervention description	Duration	Location
Baker-Henningham et al. (2009a, 2009b)	Incredible Years (IY) teacher training program	The training program focuses on	7 days of 14 classes (30–40 min),	Jamaica
	Classroom management program	Teacher–parent collaboration	And One monthly consultation in the teachers’ class	
		Building teacher–child warmth		
		Developing emotional-social skills		
		Eliminating negative behaviors		
		Classroom rules, children anger management, understanding emotions, and peer relation		
Baker-Henningham et al. (2012), Baker-Henningham and Walker, (2018)	IY program	The IY program with additional activities and roleplay, based on Jamaican teachers’ needs	8 days	Jamaica
Bloomquist et al. (1991)	Cognitive Behavior Therapy CBT-focused training for teachers, parents, and children:	The training focuses on understanding ADHD in children, problem-solving skills, teaching behavior consequences, reinforcing children’s problem solving in school	10 weeks	USA
Caldarella et al. (2018)		The program focuses on improving teachers’ implication of classroom management, including positive behavior intervention and support for strategies, social skills, and self-management	4 months	USA
Corkum et al. (2019)	web-based intervention behavior management for ADHD	The program focuses on six different ADHD aspects. The website includes a variety of materials, sessions are as follows:	6 weeks	Canada
		ADHD misconceptions effects, causes, and possible treatments, school–parent relationship, behavior plan targeting for a specific child, classroom organization, modifying classwork, and improving teacher–student relationship (TSR)		
		Addressing academic and cognitive students’ need		
		Preparing teachers to teach learning skills, self-regulation and meta-cognition, evaluate the child’s improvement, phase out the current behavior plan, and prepare follow-up plans		
		Coaching through discussion board was available		
Conroy et al. (2015)	Best In Class intervention classroom-based intervention	The intervention consists of training on setting rules, preventing challenging behavior before erupting, offering children opportunities to respond during activities, using specific praise during activities, and delivering positive feedback, followed by coaching and observation	6 h of training followed by 14- 1:30 min sessions of coaching and 30 min observation	USA
Conroy et al. (2021)	Best In Class intervention web-based intervention	One day technology training for the use of the platform and the app that is used by the teacher to record themselves in the classroom and get feedback. The eighth component of (best in class) was placed in one platform. In the coaching session, the teacher and the coach will go through the information in the model and then coaches provide feedback for the video that was sent using the app	14 coaching sessions via Zoom	USA
Downer et al. (2018)	Video feedback and consultation Behavior management strategies	The early childhood consultation model (Learning to Objectively Observe Kids (LOOK) helps teachers’ selection and behavioral strategies, using data from video feedback and validated measures and forming a social-emotional teaching strategy	Fall and spring of 2014–2015	USA
Gonzales-Ball & Bratton, (2019)		A 30-min video recorded play session was also preformed weekly on a “child of focus.”	Phase 1 was a 10-session protocol, including two full days of didactics (5 sessions), then seven weeks of 1-h group training (5 sessions)	USA
		Phase 2 training included in-class coaching		
Hickey et al. (2017)	IY TCM training + using videotape modeling	IY TCM training + using videotape modeling, role plays, and group discussion	5 sessions (one per month), and one phone call during one interval between monthly session	Ireland
Hoogendijk et al., (2018, 2020)	Key2Tech	Developing a teacher-focused coaching system (Key2Teach for primary school children) to help improve STR, with the key elements involving interaction; insight into mental representation of the STR; and polishing teachers’ interactive skills	Both phases involved 12 sessions and 2 videotaped lessons: four sessions (phase 1) and eight sessions (phase 2)	Netherlands
	Coaching in functional behavior analysis			
		Key2Tech comprised two phases and four building blocks		
		Phase 1: Offering the teacher insights into TSR using elements of functional behavior analysis		
		Phase 2: Promoting the positive interaction patterns among students and teachers		
Hutchings et al. (2013)	IY TCM training program	The training focuses on developing a better TSR, improving teachers’ strategies, including praise, reinforcement, and responsiveness, encouraging students to be ambitious, finding ways to reduce negative behavior, training children to regulate their emotional and social skills	1 day monthly for 5 months	Wales
LoCasale-Crouch et al. (2018), Williford et al. (2017)	Counseling (Banking time)	The training focus is on improving interaction quality not quantity by implementing four critical skills, observing a child's action, narrating it, accurately labeling a child’s emotions, and developing themes. The teachers interact with the child in play time and videotape the session and send it to the counselor. The counseling session focuses on problem solving based on the need of the teacher	Face to face coaching session, and a brief phone call in the alternate weeks (around 10 min play time with the child) 2–3 times weekly for eight weeks	USA
McCullough et al. (2021)	Best In Class intervention classroom-based intervention	See Conroy et al. (2015)	6 h of training followed by 14- 1:30 min sessions of coaching and 30 min observation	USA
Owens et al. (2017)	Daily report card and consultation	The program consists of a workshop and a consultation. The workshop starts with an introduction about ADHD, general classroom management, and a daily report card. Then, an interview with the teacher will be conducted regarding classroom management. Then, the teacher will learn how to develop the daily report card. Meetings with the counselor will be conducted biweekly to assess teacher performance and provide feedback, including classroom management strategies and report card graphs, as well as teachers’ values and beliefs	3-h workshop/fact sheets 6 consultation/30 min-hour consultation every other week	USA
Reinke et al. (2014)	IY TCM training Behavior management strategies	In the training, the coaches spent time in action planning and providing performance feedback on teachers’ implementation of the behavior support plans	6 days	USA
		Additionally, behavior support plans for targeted children		
Spilt et al. (2012)	Relationship-focused reflection program (RFRP)	The training starts with teacher relationship interview (TRI), then coaches will link the video observation with the interview data and provide (recommendation and strategies)	4 sessions of 45–60 min	Netherlands
Stoiber and Gettinger (2011)	Functional behavior assessment	The training contains basic concepts: conducting functional assessment/goal planning/establishing positive behavior to support PBS/applying and monitoring the PSP/reviewing and assessing results	2:30-h session followed by 2 weeks observation, functional analysis (FA) 5-h PBS developing. Implementation 8–10 Weeks	USA
Conroy et al., (2018, 2019) and Sutherland et al., (2018a, 2018b)	Best In Class intervention classroom-based intervention	See Conroy et al. (2015)	One day teachers’ workshop/14 coaching weeks/one weekly session	USA
Sutherland et al. (2020)	Best In Class intervention classroom-based intervention	See Conroy et al. (2015)	6 h of training followed by 14- 1:30 min sessions of coaching and 30	USA
Vancraeyveldt et al., (2015a, 2015b)	Playing together session adapted from Banking time	Teachers were given a manual and watched a DVD demonstrating good and bad strategies. They were also given behavior medication strategies and learnt how to improve teacher interaction with children during the play session by observing and narrating on a child's action, describing child’s emotions, and developing themes. The teachers would interact with the child in playtime session and send it to the counselor. The counseling session focuses on problem solving based on the need of the teacher	7/2 h session	Belgium
Veenman et al., (2017, 2019)	Positivity and Rules Program (Manual)	A manual contained ADHD symptoms, evidence-based classroom strategies, and contingency management. The most important factor in the training was how to implement the intervention systematically and precisely	6 weeks	Netherlands

### Data Extraction and Statistical Analysis

Both authors performed data extraction at an independent level and agreed on the data extraction. The standardized mean difference (SMD) represented the mean change pre-to-post-treatment for the intervention arm minus the mean change pre-to-post-treatment for the control arm divided by the pooled pre-test standard deviation with a bias adjustment. The researchers performed the calculations using the Review Manager (RevMan) computer program, Version5.3. Copenhagen: The Nordic Cochrane Centre, The Cochrane Collaboration group (Cochrane Collaboration, 2014). This paper used the Inverse-Variance IV method and a random-effects model due to the integral heterogeneity of studies, calculating the I^2^ statistics, a posteriori, to give an estimation of between-trial SMD heterogeneity (Field & Gillett, 2010).

### Coding Procedure

The first author coded all the data and the last author verified the coding. The study codes consisted of (a) children’s selection method and by whom, (b) study design, (c) type of control condition, (d) number of participants (whether they were teachers, children, or both in intervention and control arm), (e) age of children, (f) teachers’ years of experience, (g) female percentage for teachers, (h) male percentage for children (see Table 4). The intervention data coding included a description of the intervention, including type, duration, and geographic location (see Table 5).

**Table 4:** Details of the specific measures used for each analysis

Study	Teachers’ outcomes	Children’s outcomes					
	CL	TAS	CON	EBP	PRO	ADHD	CP^b^
Baker-Henningham et al. (2009a)							
MPROX				SDQ	SDQ	SDQ	
PBLIND							
Baker-Henningham et al. (2009b)							
MPROX							
PBLIND	TPOT						
Baker-Henningham et al. (2012)							
MPROX				SDQ SESBI	SDQ	CTRS	
PBLIND							DPICS
Baker-Henningham and Walker, (2018)							
MPROX		MOOSES DPICS					
PBLIND							
Bloomquist et al. (1991)							
MPROX				CTRS	WMCSC	CTRS	
PBLIND							
Caldarella et al. (2018)							
MPROX				SSBS-2	SSBS-2		
PBLIND							
Conroy et al. (2015)							
MPROX		TCIDOS		TCIDOS*			
PBLIN							TCIDOS*
Corkum et al. (2019)							
MPROX						CTRS	
PBLIND							
Downer et al. (2018)							
MPROX		CMSQ					
PBLIND							
Gonzales-Ball & Bratton, (2019)							
MPROX				CTRF			
PBLIND							
Hickey et al. (2017)							
MPROX				SDQ	SDQ	SDQ	
PBLIND		TPOT					TPOT
Hoogendijk et al. (2020)							
MPROX	STRS		STRS				
PBLIND		CLASS					
Hutchings et al. (2013)							
MPROX							
PBLIND					TPOT		
LoCasale-Crouch et al. (2018)							
MPROX	STRS		STRS				
PBLIND							
Reinke et al. (2014)							
MPROX				TOCA-C	TOCA-C		
PBLIND		MOOSES					
Spilt et al. (2012)							
MPROX	STRS		STRS				
PBLIND							
Stoiber and Gettinger (2011)							
MPROX				SCP	SCP		
PBLIND		OREVS					CCOF
Sutherland et al. (2018a)							
MPROX	STRS		STRS	SSIS-RS			
PBLIND							
Sutherland et al. (2020)							
MPROX	STRS	CLASS	STRS	TCIDOS	SSIS		
PBLIND							
Vancraeyveldt et al. (2015a)							
MPROX				PBQ			
PBLIND							
Williford et al. (2017)							
MPROX				ECBI + SESBI–R			
PBLIND							

*TCIDO was used in two analyses as it was the only conduct problem measure in Conroy’s study, therefore it was counted as MPROX & PBLIND

ADHD/C, Attention Deficit Hyperactivity Disorder Combined; CCOF, Classroom Competence Observation Form (Stoiber, 2004); Cl, Closeness; CLASS, Classroom Assessment Scoring System; CMSQ, Classroom Management Strategies Questionnaire (Webster-Stratton, 2012); CON, Conflict; CP, Conduct Problems; CTRF, Caregiver Teacher Report Form (Achenbach & Rescorla, 2000); CTRS, Conners Teacher Rating Scale (Goyette et al., 1978); DPICS, Dyadic Parent Child Inter-active Coding System (Eyberg & Robinson, 1981); EBP, Externalizing Behavior Problems; ECBI, Eyberg & Pincus (Eyberg, 1999); MOOSES, Multi-Option Observation System for Experimental Studies (Tapp et al., 1995); MPROX, Most Proximal; OREVS, Observer Rating of Ecobehavioral Variables Scale (Chandler et al., 1999); PBLIND, Probably Blinded; PBQ, Preschool Behavior Questionnaire (Behar, 1977); PRO, Prosocial; SCP, Social Competence Performance Checklist (Stoiber, 2004); SDQ, Strengths and Difficulties Questionnaire (Goodman & Goodman, 2009); SESBI, Sutter–Eyberg Student Behavior Inventory (Rayfield et al., 1998); SSBS-2, School Social Behavior Scales–Second Edition (Merrell & Gimpel, 1998); SSIS, Social Skills Improvement System (Gresham & Elliott, 2007); STRS, Student–Teacher Relationship Scale (Pianta, 2001); TAS, Teacher use of Appropriate Strategies; TCIDOS, Teacher–Child Interaction Direct Observation System (Sutherland et al., 2013); TOCA-C, Teacher Observation of Classroom Adaptation–Checklist (Koth et al., 2009); TPOT, Teacher–Pupil Observation Tool (Martin et al., 2010); WMCSC, Walker-McConnell Scale of Social Competence & School Adjustment (Walker & McConnell, 1988)

^a^ MPROX; ^b^ PBLIND

**Table 5:** Summary of the forest plots and statistical data from

Outcome	SMD	(95% CI)	*P* value	Heterogeneity *I*^2^ (%)	$\chi^{2}$	*P* value
Teachers’ closeness	0.48	0.15–0.81	< 0.0001	75	19.73	0.001
Teachers conflict	0.19	0.05–0.34	0.009	0	3.80	0.43
Techers’ use of appropriate strategies	0.71	0.29–1.14	0.001	78	31.17	< 0.0001
Externalizing behavior MPROX	0.41	0.25–0.56	< 0.00001	48	21.25	0.03
Conduct problems PBLIND	0.33	0.04, 0.71	0.001	78	16.07	0.003
Prosocial behavior	0.46	0.28–0.64	< 0.00001	33	11.89	0.16
ADHD/C	0.47	0.3–0.65	< 0.00001	0	1.44	0.84

Statistical data included mean standard deviation and the number of participants in both arms. Eleven studies had a lack of sufficient information (e.g., blinding) and statistical information (e.g., standard deviation, mean), so the first author contacted the corresponding authors. The authors of four of these studies emailed this paper’s researchers with the missing data within the agreed time window (i.e., three reminders). In some cases, this paper estimated missing data using the method described by Hozo et al. (2005) to calculate the mean and estimate the standard deviation (Wan et al., 2014). Missing data for three studies meant that it was impossible to obtain or calculate an effect size; these studies were included in the review but excluded from the meta-analysis.

### Methodological Quality Assessment

**Fig. 2:**
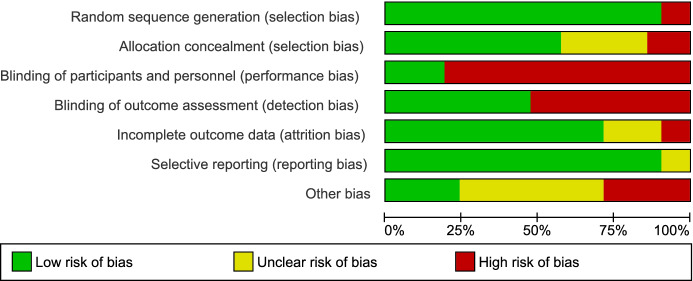
Risk of bias for summary for RCTs

Two reviewers (RD and DD) conducted the quality assessment by evaluating the quality of data in the included studies, using the Cochrane group Risk of Bias-2 (RoB-2) to assess the overall quality of the included RCTs (Higgins et al., 2011). Agreement between the two authors was 90% and any disagreements were resolved between the authors without the need for a third party. A high percentage of studies introduced some theoretical risk of selection bias since the nature of studies that included teachers and their students lacked blinding. However, adequate randomization across all studies meant that the overall risk of selection bias was low, while an adequate description of the study results reduced reporting bias across all studies. Unfortunately, the lack of a double-blinded approach in most studies introduced a higher risk of performance bias. The overall and study-level bias of the included RCTs is reported in Fig. 2.

### Outcomes Coding

The current analysis aimed to increase analytical robustness by considering outcome domains that included at least five studies, as recommended by Daley et al. (2014), Faraone et al. (2021), Higgins et al. (2019), Sonuga-Barke et al. (2013). The authors considered different perspectives, including the most proximal view (MPROX), which represents the view of the person closest to the receipt of treatment, or the probably blinded view of a person unaware of treatment allocation (PBLIND) (Daley et al., 2014; Sonuga-Barke et al., 2013). In the present study, PBLIND outcomes were only available for conduct problems. The researchers categorized outcomes as teacher-focused or child-focused outcomes.

#### Measures for Teacher-Focused Outcomes

There were three outcomes for teachers: warmth and closeness, teacher–child conflict, and teachers’ use of positive strategies. Other than one study that measured warmth using the Teacher–Pupil Observation Tool (TPOT), all studies in the warmth/closeness domain used the same measure, Student–Teacher Relationship Scale (STRS). All of the studies that comprised the teacher–child conflict outcome were based on a single measure, STRS. The studies for the outcome of teachers’ use of positive strategies were heterogeneous and included papers that used different measures for teachers’ positive behavior, such as classroom management, general praise, responsive behavior, and classroom instructional support. The teacher well-being outcome was not included because there were insufficient included studies to measure it.

#### Measures for Child-Focused Outcomes

This outcome had four domains, externalizing problems, prosocial behaviors, ADHD symptoms, and conduct problems. The externalizing behavior problems domain was heterogeneous and comprised studies that measured oppositional behavior, challenging behavior, and conduct behavior, using a variety of measures as listed in Table 4. For prosocial behavior, the included studies measured peer relationships, prosocial skills, and social behavior using a range of measures as listed in Table 4. For ADHD outcome behavior, the researchers grouped hyperactivity symptoms and inattention symptoms together for some studies if they were listed separately. The included studies in this domain used Strengths and Difficulties Questionnaire (SDQ) and Conners Teacher Rating Scale (CTRS) measures. Finally, the conduct problem PBLIND outcome used observational measures to assess aggression and conduct problems. See Table 4 for more information about the measures.

### Analysis

The researchers conducted the current analysis using a random-effects model by measuring the standard mean difference (SDM) between the intervention and control groups. Results of the meta-analysis for each teacher and child outcome are reported in Table 3.

## Results

### Teacher Outcomes

**Fig. 3:**
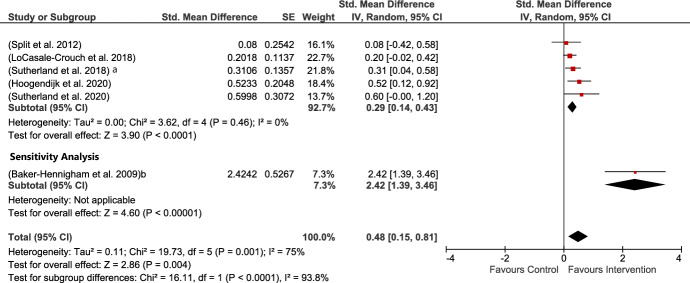
Teacher-child closeness MPROX

Six studies provided measures of teachers’ closeness toward the child. All were MPROX except for Baker-Henningham (b) (Baker-Henningham et al., 2009b). The present analysis revealed a moderate but significant result concerning the impact of interventions on teacher–child closeness. Heterogeneity among the included studies was large and significant. The SDM in this analysis was influenced by Baker-Henningham (b) who reported an SMD of 2.42. Sensitivity analysis removing this study still provided a small but significant result in favor of intervention (SMD of 0.29) and 0% heterogeneity because the remaining studies all used the same instrument (see Fig. 3). Heterogeneity is the clinical variability within the various samples, methodological variability across the various included studies, and statistical variability among the included studies in an outcome.

**Fig. 4:**
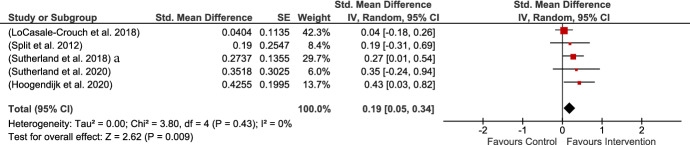
Teacher-child conflict MPROX

Five studies provided measures of teacher–child conflict based on teacher ratings, all of which were MPROX. The current analysis revealed a small but significant result of intervention on teacher–child conflict. Heterogeneity among the included studies was not significant as all the studies used the same measure (see Fig. 4).

**Fig. 5:**
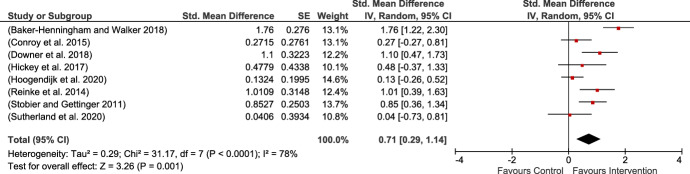
Teachers’ use of appropriate strategies (mixture between MPROX and PBLIND)

Eight studies explored teachers’ use of appropriate strategies. All observational measures except for Downer et al. (2018) consisted of rating scales. Four studies (Baker-Henningham & Walker, 2018; Conroy et al., 2015; Downer et al., 2018; Sutherland et al., 2020) were MPROX and the other four (Hickey et al., 2017; Hoogendijk et al., 2020; Reinke et al., 2014; Stoiber & Gettinger, 2011) were PBLIND. There was an insufficient number of blinded studies that represent the view of the person closest to the person unaware of treatment allocation to conduct a separate PBLIND outcome. Heterogeneity among the included studies was large and significant (see Fig. 5).

### Child Outcomes

**Fig. 6:**
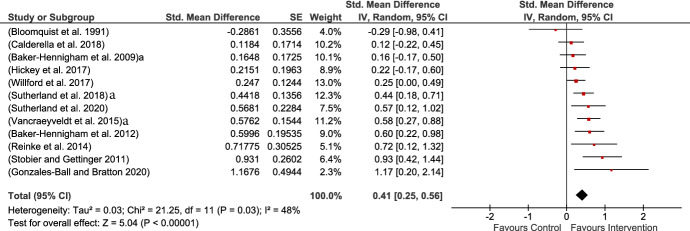
Externalizing behaviors MPROX

Thirteen studies included measures of child’s externalizing behavior. All of them were teacher-rated, and MPROX except for Conroy study that used an observational measure (Conroy et al., 2015). The current analysis revealed a moderate but significant impact of intervention on reducing externalizing behavior. Heterogeneity in the analysis was small but significant (Fig. 6).

**Fig. 7:**
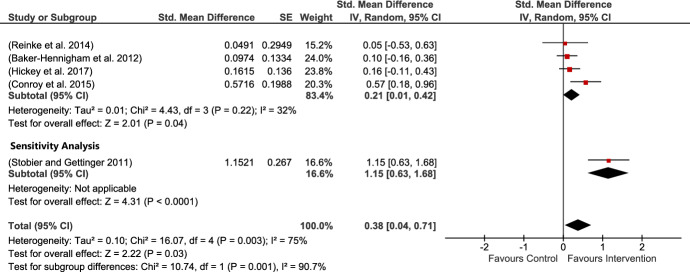
Conduct problem PBLIND

Five studies provided PBLIND measures of child conduct problems in the form of observational measures. The current analysis revealed a large and significant impact of interventions on child conduct problems. There was, however, considerable heterogeneity, which was significant. The SDM in this analysis was influenced by Stoiber’s study (Stoiber & Gettinger, 2011), who reported an SMD of 1.15. Sensitivity analysis removing this study still provided a small but significant SMD of 0.21 (see Fig. 7).

**Fig. 8:**
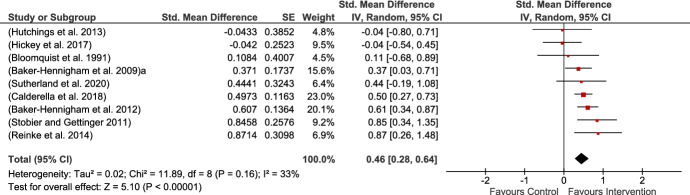
Prosocial behavior MPROX

Nine studies provided measures of child prosocial behavior, eight of which provided teacher ratings, all of which were MPROX, while one study provided blinded observational scores (Hutchings et al., 2013). There was a moderate but significant result of the impact of interventions on prosocial behavior and heterogeneity was low and nonsignificant (see Fig. 8).

**Fig. 9:**
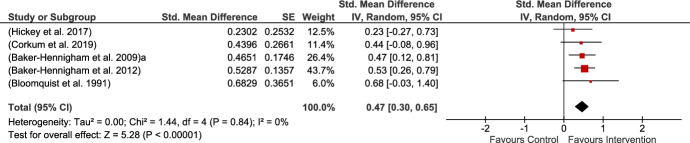
ADHD/C MPROX

Six studies provided measures of child ADHD combined symptoms, all of which were MPROX and teacher-rated. The results indicated a moderate but significant impact of the intervention on ADHD symptoms in children. Heterogeneity was 0% in the analysis, perhaps since the majority of the studies used the same measure (see Fig. 9).

## Discussion

The current meta-analysis of RCTs investigated the efficacy of providing teacher support for children with externalizing problems, based on both teacher and child-focused outcomes. In total, 22 studies contributed to the meta-analysis, which included 861 teachers and 1841 children across intervention and control arms. Where possible, the results were reviewed by examining two different viewpoints including the MPROX and a PBLIND (Daley et al., 2014; Sonuga-Barke et al., 2013). Due to the variability of intervention targets, children’s ages, and outcomes, considerable heterogeneity was evident in the meta-analysis except where the same measures were used in all or most studies.

Both teacher and child outcomes were included in the current analysis, which found significant and moderate teacher-mediated intervention-related improvements for teacher–child closeness. Teacher use of appropriate strategies was a mixture of MPROX (provided by individuals who are aware of intervention allocation) and PBLIND outcomes (provided by individuals who are unaware of intervention allocation based on questionnaires and observations provided by individuals). The results found large and significant improvements in favor of intervention for teachers’ use of classroom management strategies, providing general praise to students, and utilizing responsive behavior in the classroom, in contrast to the smaller but significant results for the intervention that led to reduced conflict between teachers and children with externalizing behavior. Taken together, these findings support the positive impact of interventions on teachers’ skills development and relationships with children, although the results for teacher outcomes lacked confirmation from blinded outcomes.

The current meta-analysis also highlighted intervention-related improvement in child behavior problems. In particular, the results revealed a moderate but significant reduction in externalizing behavior problems and ADHD symptoms in children. Moreover, the results also found a moderate and significant increase in children’s prosocial behavior as a result of teacher-mediated intervention in the included studies.

Blinded evidence for the efficacy of teacher-mediated interventions was explored in the current analysis on child conduct problems using outcomes provided by individuals who are unaware of intervention allocation. This outcome was the only blinded analysis in the current meta-analysis and confirmed the impact of interventions on blinded teacher reports of child conduct behavior. This finding highlights the need for more blinded outcomes to further explore and test the effectiveness of teachers’ interventions beyond unblinded teacher ratings, in line with recommendations by Daley et al. (2014).

Results from the current meta-analysis suggest that teacher-mediated interventions demonstrated an improvement in conduct problems using PBLIND measures. Significant improvements were also demonstrated by MPROX measures for children’s ADHD-related symptoms and prosocial behavior. These results are in line with the results of Daley et al. (2014), which reported significant MPROX and PBLIND improvements in conduct problems; but only MPROX improvements in ADHD-related symptoms for parent-mediated interventions for children with ADHD.

Another meta-analysis (Iznardo et al., 2020) was conducted to examine the effectiveness of more focused daily report cards interventions in reducing ADHD symptoms rated by teachers also reported a reduction in conduct problems for children with ADHD using MPROX (rating scales) and PBLIND (observational measures). In fact, the PBLIND outcomes were found to be more sensitive in measuring ADHD symptom change compared to rating scales although this meta-analytic study included non-RCTs and there were less than five studies in the PBLIND analysis (Iznardo et al., 2020).

The current meta-analysis also found a significant reduction in conduct problems and ADHD symptoms with larger effect sizes than a previous meta-analysis from Stoltz et al. (2012). Stoltz et al. investigated the efficacy of school-based interventions on school-aged children with externalizing behavior from two different perspectives. The first was solely child-focused interventions. The second included other intervention targets in addition to a focus on the child, such as modifying the school environment and direct parent support. Both perspectives (the child-focused-only intervention and the intervention that included both child and other elements) demonstrated a reduction in externalizing behavior with SMD 0.30 and 95% CI = 0.14 − 0.46 for child-focused intervention and SMD 0.30 and 95% CI = 0.04 − 0.56 for the child-focused and other components intervention (Stoltz et al., 2012).

ADHD symptoms in particular can negatively impact the teacher–student relationship (Rogers et al., 2016). Enhancing warmth and closeness may be potentially beneficial to children, as Wang et al. (2016) demonstrated in their study using measures of closeness and conflict. The study revealed that children whose emotional relationship with their teachers was high in closeness and low in conflict had better peer relationships and fewer emotional/internalizing problems (Wang et al., 2016). In contrast, the results of the present meta-analysis demonstrated a small but significant enhancement in closeness and reduction of conflict in favor of intervention. These improvements may have an additional impact on children’s outcomes beyond what could be identified in the current analysis.

The current results highlight that little is known about the impact of teacher-mediated interventions on teachers’ self-efficacy and well-being. The number of studies that measured teachers’ stress levels or self-efficacy in the current analysis was insufficient to draw conclusions about these dependent variables. This was surprising and highlights a gap in the current literature since several studies have demonstrated the relationship between children’s level of externalizing behaviors and teachers’ level of confidence in classroom management (Arbuckle & Little, 2004; Collie et al., 2012; Stephenson et al., 2000). School responsibilities and workload can also cause stress and negative emotions in teachers (Fernet et al., 2012; Skaalvik & Skaalvik, 2011). Liu and Onwuegbuzie (2012) also found that disruptive behavior in the classroom is one of the primary causes of teachers’ stress (Liu & Onwuegbuzie, 2012). Therefore, future studies should investigate the impact of teacher-mediated interventions on teacher stress and self-efficacy in greater detail. The aim would be to either confirm that current interventions also target teacher well-being, or potentially highlight the need for specific interventions that target these important outcomes.

The interventions included in the current meta-analysis were predominantly delivered face to face and only two online interventions were included in the review. Given the considerable work-related pressure that teachers encounter and the financial limitations they experience when taking time away from the classroom to enhance their skill set, this finding was also surprising. Future research should expand the evaluation of digital interventions for teachers to widen participation and reduce barriers to engagement, especially considering the additional limitations that have been imposed during the COVID-19 pandemic.

To the best of our knowledge, this study represents the first meta-analysis of teacher-mediated interventions for children with externalizing behaviors. The current analysis focused on peer-reviewed RCTs only and involved a systematic search conducted across five databases. Moreover, the current analysis focused on two different sets of outcomes including teacher and child, as well as two levels of reporting, MPROX and PBLIND. The outcomes were informed by the recommended number of studies necessary in a meta-analysis (Daley et al., 2014; Faraone et al., 2021; Higgins et al., 2019; Sonuga-Barke et al., 2013). However, this meta-analysis was limited by the lack of data on teachers’ outcomes in the included studies and the inability to confirm any of the teacher outcomes using PBLIND measures.

A second limitation was that an investigation of the long-term impact of teacher intervention was not possible due to the lack of sufficient data since many studies did not include long-term follow-up data, meaning that the long-term extent of behavior change remains unclear. A third limitation was that the total number of teachers involved could not be precisely measured because some studies that focused primarily on child outcomes did not indicate the number of teachers involved in the study. Fourth, we included studies in the ADHD outcome if they had an ADHD symptoms result, but we are unaware of comorbidity.

Fifth, it was difficult to explore publication bias because this review only included published papers. However, the authors generated and inspected funnel plots in line with recommendations from the Cochrane group (Higgins et al., 2019), and restricted the interpretation of funnel plots for outcomes with 10 or more studies and where there was heterogeneity in measures only; all of which made it impossible to comment on publication bias within the current analysis.

Sixth, while some scholars recommend the inclusion of unpublished studies in meta-analyses; the restriction of the analysis to published studies was to ensure greater methodological rigor in the analysis and enhance the validity of the findings and recommendations. Finally, the present study calculated the effect sizes using shifting units of analysis, as is standard practice within the Cochrane group (Higgins et al., 2019). However, this method can increase the number of statistical tests run (Pigott & Polanin, 2020). Using a different approach such as robust variance estimation or multi-level modeling can adjust the effect size and increase independency (Tipton et al., 2019; Van den Noortgate et al., 2015) although it was not suitable for this particular analysis.

This meta-analysis, focusing on both teacher and child outcomes, aimed to explore the potential of interventions for supporting teachers of children with externalizing problems in school. The results of this study indicate that addressing externalizing problems in children using teacher-mediated intervention was beneficial for both teachers and children. Moreover, the current results suggest that future research should examine the impact of teacher-mediated interventions on teachers’ well-being and self-efficacy. It would also be important in the future for trials to include more PBLIND measures so that current MPROX findings could be confirmed using PBLIND outcomes.

## Data Availability

Data can be obtained from the first author following completion of her PhD.
